# Factors Affecting Length of Stays in the Emergency Department of a Teaching Hospital: A Retrospective Study From Najran, Saudi Arabia

**DOI:** 10.7759/cureus.64684

**Published:** 2024-07-16

**Authors:** Ahmed M Badheeb, Mohammed A Almutairi, Abbas H Almakrami, Abdelaziz A Aman, Ali Dhafer Al-Swedan, Khalil Alrajjal, Islam A Seada, Abdullah Abu Bakar, Samer Alkarak, Faisal Ahmed, Awadalla Babiker, Sindhu Susheer, Mohamed Badheeb, Mofeed Almutairi, Hamoud Y Obied

**Affiliations:** 1 Oncology, King Khalid Hospital-Oncology Center, Najran, SAU; 2 Internal Medicine, King Khalid Hospital, Najran, SAU; 3 Endocrinology, King Khalid Hospital, Najran, SAU; 4 Internal Medicine/Endocrine and Diabetes, King Khalid Hospital, Najran, SAU; 5 Infectious Disease, King Khalid Hospital, Najran, SAU; 6 Cardiothoracic Surgery, King Khalid Hospital, Najran, SAU; 7 Ophthalmology, King Khalid Hospital, Najran, SAU; 8 General Surgery, King Khalid Hospital, Najran, SAU; 9 Urology, Ibb University, Ibb, YEM; 10 Quality and Patient Safety, King Khalid Hospital, Najran, SAU; 11 Internal Medicine, Yale New Haven Health, Bridgeport Hospital, Bridgeport, USA; 12 Surgery, Najran University, Najran, SAU; 13 Cardiac Surgery, King Khalid Hospital, Najran, SAU

**Keywords:** saudi arabia, najran, length of stay, canadian triage and acuity scale delayed admission, emergency department

## Abstract

Background: Reducing the frequency of emergency department (ED) patient visits for treatment, particularly in urgent instances, is a global healthcare objective. Additionally, a more extended stay in the ED can harm a patient's prognosis during later hospitalization. This study aims to investigate the factors affecting the length of stay in the ED in a teaching hospital.

Methods: A retrospective chart review study was done between January 1, 2021, and February 31, 2021, involving 122 adult patients who had delayed ED visits to King Khalid Hospital in Najran, Saudi Arabia. Data on the patient's characteristics, visit time, and the causes for the delay based on the Canadian Triage and Acuity Scale (CTAS) were gathered and analyzed. Factors associated with more than six hours of delay were investigated in a univariate analysis.

Result: The mean age was 52.3 ±13.5 years, and 42 (34.4%) were more than 65 years of age. More than half of the study population were female (n=66; 54.1%). Most delays occurred among CTAS 4 and 5 cases (47.5%), and 22 (18.0%) occurred during holidays. The mean delay time was 6.1 ±1.8 hours. The leading delay causes were multiple consultations with further investigations (37.7%) and conflict between the teams (36.1%). In univariate analysis, ED visiting at holiday time (OR: 0.14; 95% CI: 0.04-0.40, p <0.001) and CTAS 4 and 5 (OR: 2.22; 95% CI: 0.95-5.30, p = 0.003) significantly had more delay. Factors associated with delay in univariate analysis were multiple consultations with further investigations (OR: 2.82; 95% CI: 1.32-6.26, p = 0.013), various assessments in different ED areas with a late arrival of the specialist (OR: 0.43; 95% CI: 0.20-0.91, p = 0.042), and conflict between the teams (OR: 2.50; 95% CI: 1.17-5.54, p = 0.031).

Conclusion: In this study, multiple assessments in different ED areas and conflict between the teams were the main factors that caused delays in ED. Implementing a timeframe monitoring system for consultations while emphasizing accelerated decision-making and disposition for patients and understanding teamwork collaboration may reduce patients' length of stay in the ED. Implementing these strategies and evaluating their impact on the length of stay in the ED requires further investigation.

## Introduction

Overcrowding in emergency departments (ED) causes more extended hospital stays and worse treatment quality, which are significant concerns for public healthcare systems worldwide [1]. Longer stays in the ED have become a key performance indicator for decision-makers to monitor and adjust performance [1, 2]. Long lengths of stay in the ED can lead to patient safety issues, delayed care, dissatisfaction, medical mistakes, and weariness among ED healthcare workers [2, 3]. Various strategies, including team-based triage, fast-tracking, laboratory analysis, and X-ray imaging requested by nursing staff have been suggested to reduce the length of stay in the ED [3].

There are numerous causes of ED lengths of stay; for example, outpatient treatment for cases with non-urgent conditions, under triage, underestimation of comorbidities, fragility, crowding, insufficient inpatient beds, a higher number of specialties consultations, a conflict between the team, and need for ICU admission [2,4-6]. Other variables related to protracted lengths of stay include inadvertent physician repeat orders, needless blood chemistry tests, radiological imaging, and admissions to the ED on weekends and late afternoon or night hours [2,7]. A Dutch study indicated that organizational characteristics such as more consultations, testing in the ED, and lower physician seniority were associated with prolonged ED lengths of stay [8]. Other studies have found that patient ethnicity, the timing of ED presentation, the season of ED presentation, old age, insurance status, complexity of the patient's disease, and ED physician treatment style all impacted ED lengths of stay [9,10].

While various studies have examined lengths of stay concerning the initial ED diagnosis, only a few studies have been performed in Saudi Arabia [5, 11]. A previous report from Saudi Arabia mentioned that demographic factors such as age, gender, shift time, disposition status, and nonurgent Canadian Triage and Acuity Scale (CTAS) cases were significant factors that must be considered to reduce patients' length of stay in ED [12]. However, previous reports on time objectives in EDs have produced conflicting findings, with both good and adverse outcomes beyond their intended impact. This study aims to investigate the factors affecting the length of stay in the ED in a teaching hospital in Najran, Saudi Arabia. The current study would close that gap, provide valuable evidence, and add to the current knowledge base concerning the relationship between ED crowding status and patient outcomes.

## Materials and methods

Study design

**Figure 1:**
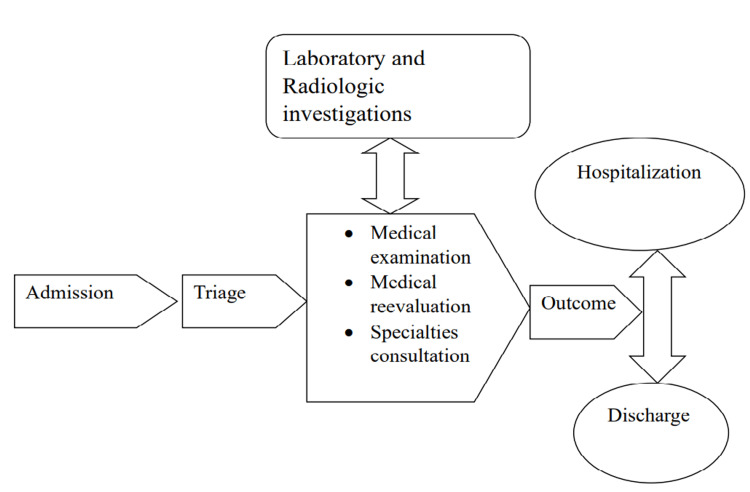
The traditional clinical-care path in the emergency department.

A retrospective chart review study was done between January 1, 2021, and February 31, 2021, involving 122 adult patients who had delayed ED visits to King Khalid Hospital in Najran, Saudi Arabia, an academic institution with an annual emergency department census of 16,191 patients during 2021 [13]. This study was approved by the Ethics Research Committees of King Khalid Hospital (Code: KACST, KSA: H-I1-N-089) in compliance with the ethical standards outlined in the Declaration of Helsinki. Owing to the study's retrospective nature, written informed consent from the included patients was not required. The ED is open 24 hours a day, seven days a week, and is staffed by board-certified emergency physicians, residents, interns, and registered nurses. The six-hour cut-off was selected based on the department's policy definition for prolonged ED length of stay. The traditional clinical care path in the emergency department is illustrated in Figure 1.

The ED visits were classified into Canadian triage acuity scale (CTAS) levels, representing patients who required resuscitation (Triage 1), emergent (Triage 2; necessitates examination within 1-14 minutes), urgent (Triage 3; necessitate treatment within 60 minutes), less-urgent (Triage 4; must be considered within 1-2 hours), and non-urgent cases (Triage 5; a delay of up to 24 hours would not significantly impact the clinical state, and the patient may be sent to the appropriate alternative specialist), based on the triage nurse's assessment of the patient's need for prompt examination, stabilization, or treatment [14]. The collected data include the patient's characteristics, including age and gender, CTAS levels, delayed type (before and after admission), time of ED visit, reasons for delay, time, and conflict between times. Consultation status was recorded as no consultation, consultation, or multidisciplinary resuscitation. In the case of one or multiple consultations, the observer registered the number of consultations and consultation reasons, consulted specialties, and requested specialist(s). The main outcome was investigating the factors associated with ED delay. The secondary outcome was investigating the factors associated with more than six hours of ED delay.

Statistical analysis

Continuous data were presented as mean and standard deviation (SD) if normally distributed and median (interquartile range: IQR) if skewed. Categorical data were presented as numbers (%). Categorical variables were analyzed using the Chi-square test or Fisher's exact test, whereas continuous variables were evaluated using the Student's t-test or the Mann-Whitney U test. The odds ratios and 95% confidence intervals were calculated to assess the strength of the associations. Statistical significance was set at p < 0.05. IBM SPSS version 25 software (IBM Corp., Armonk, New York) was used for statistical analyses. Statistical significance was defined as a two-tailed P-value < 0.05.

## Results

The mean age was 52.3 ±13.5 years (19.0-71.0 years), and 42 (34.4%) were more than 65 years old. Most cases were female, 66 (54.1%); 22 (18.0%) delays occurred during holidays, and 62 (50.8%) occurred before the decision of hospital admission. Most delays (n= 58, 47.5%) occurred in nonurgent cases (Triage 4 and 5). Delays in other CTAS levels Triage 3, Triage 2, and Triage one occurred in 36 (29.5%), 24 (19.7%), and 4 (3.3%), respectively (Table 1).

**Table 1:** Characteristics of the patients who experienced delays in the emergency department

Variables	N (%)
Age (year), Mean ±SD	52.3 ±13.5
Age groups	
Less than 65 years	80 (65.6%)
More than 65 years	42 (34.4%)
Gender	
Male	56 (45.9%)
Female	66 (54.1%)
Time of delay	
During usual time	100 (82.0%)
During holiday	22 (18.0%)
Delay time based on admission	
Before the decision of hospital admission	62 (50.8%)
After the decision of hospital admission	60 (49.2%)
Canadian Triage and Acuity Scale	
Triage 4 and 5	58 (47.5%)
Triage 3 or urgent visit	24 (19.7%)
Triage 2, or emergency visit	36 (29.5%)
Triage 1 (resuscitation)	4 (3.3%)

The mean delay time was 6.1 ±1.8 hours (4.0-10.0 hours). The leading causes of delay were multiple consultations with further investigations advised in 46 (37.7%) cases and conflict between the teams in 44 (36.1%) cases (Table 2). The proportion of agreement between emergency physicians' and consultants' opinions on admission needs was 63.9.1%.76 (62.3%) of patients received one consultation and 46 (37.7%) of patients received multiple consultations, with nine (7.37%) perceived as difficult by emergency physicians. Additionally, in the ER setting, 37.7% of cases received appropriate and important further investigations, while 9.83% were deemed inappropriate or unimportant.

**Table 2:** Causes of delay in the emergency department and delay in time subgroup.

Variables	N (%)
Multiple consultations with further investigations advised	46 (37.7%)
Critical care management	24 (19.7%)
Multiple assessments in different ED areas followed by specialist late arrival	48 (39.3%)
Conflict between the teams	44 (36.1%)
Delay time (hours), Mean ±SD	6.1 ±1.8
Delay in time subgroup	
≤ 6 hours	56 (45.9%)
> 6 hours	66 (54.1%)

Factors associated with more than six hours of ED delay

In univariate analysis, ED visiting at holiday time (OR: 0.14; 95% CI: 0.04-0.40, p <0.001) and CTAS 4 and 5 (OR: 2.22; 95% CI: 0.95-5.30, p=0.003) significantly had more delay. Factors associated with delay in univariate analysis were multiple consultations with further investigations advised (OR: 2.82; 95% CI: 1.32-6.26, p=0.013), various assessments in different ED areas with a late arrival of the specialist (OR:0.43; 95% CI: 0.20-0.91, p=0.042), and conflict between the teams (OR: 2.50; 95% CI: 1.17-5.54, p=0.031) (Table 3).

**Table 3:** Factors associated with more than six hours of emergency department delay

Variables	Subgroup	Less than 6 hours	More than 6 hours	OR (95% CI)	p-value
Age (year)	Mean ±SD	52.2 (13.2)	52.4 ±13.9	1.00 (0.97-1.03)	0.940
Gender	Male	26 (46.4)	30 (45.5)	Reference group	1.000
	Female	30 (53.6)	36 (54.5)	1.04 (0.51-2.13)	
Delay time based on admission	Before admission	32 (57.1)	30 (45.5)	Reference group	0.269
	After admission	24 (42.9)	36 (54.5)	1.60 (0.78-3.30)	
Time of delay	Usual time	38 (67.9)	62 (93.9)	Reference group	<0.001
	Holiday time	18 (32.1)	4 (6.1)	0.14 (0.04-0.40)	
Canadian Triage and Acuity Scale	Triage 4 and 5	18 (32.1)	18 (27.3)	Reference group	0.003
	Triage 3	18 (32.1)	40 (60.6)	2.22 (0.95-5.30)	
	Triage 2	18 (32.1)	6 (9.1)	0.33 (0.10-1.00)	
	Triage 1	2 (3.6)	2 (3.0)	1.00 (0.11-9.09)	
Multiple consultations with further investigations advised	No	42 (75.0)	34 (51.5)	Reference group	0.013
	Yes	14 (25.0)	32 (48.5)	2.82 (1.32-6.26)	
Critical care management	No	48 (85.7)	50 (75.8)	Reference group	0.250
	Yes	8 (14.3)	16 (24.2)	1.92 (0.77-5.12)	
Multiple assessments in different ED areas with specialist late arrival	No	28 (50.0)	46 (69.7)	Reference group	0.042
	Yes	28 (50.0)	20 (30.3)	0.43 (0.20-0.91)	
Conflict between the teams	No	42 (75.0)	36 (54.5)	Reference group	0.031
	Yes	14 (25.0)	30 (45.5)	2.50 (1.17-5.54)	

## Discussion

Numerous studies have shown that non-urgent visits to EDs contribute to congestion and an increased duration of stay [14,15]. Others, however, argue that non-urgent patients arriving at the ED are not to blame for congestion and a longer duration of stay [16]. Our result shows that 29.5% of patients were triaged as urgent and 47.5% as non-urgent. Similar to this study's findings, other research found that a substantial proportion (>80%) of non-urgent patients were addressed in emergency departments [17,18]. Additionally, our result was in agreement with other reports from western Saudi Arabia and Princess Nourah Bint Abdulrahman University, which reported that non-urgent visits accounted for 78.5% and 61.4% of non-life-threatening cases, respectively [14,19]. In contrast, research done in South Africa by Mashao et al. found that 66% were triaged as urgent (urgent and highly urgent), whereas 34% were non-urgent [20]. Additionally, our result found that patients who triage as non-urgent were a predictor for ED delay in univariate analysis (OR: 2.22 and p=0.003). Hospitals should set up a distinct ED for less-urgent and non-urgent problems, which general or family doctors should address. It enables prompt care without jeopardizing ED services since general physicians may manage patients with limited resources without raising re-attendance rates.

In our study, the mean time from decision to disposition to actual discharge or transfer from the ED was 6.1 ±1.8 hours, and 66 (54.1%) cases were longer than six hours. Similar to our result, other studies reported a mean length of stay of four hours, up to six hours [21, 22]. In contrast, research done in South Africa by Mashao et al. found that the mean overall time from when patients reported to reception to being transferred or discharged from the ED was 73 hours [20].

In this study, most cases were adults aged less than 65 years (65.6%). The result was similar to previous reports from Saudi Arabia. For example, Alnasser et al. mentioned that individuals between 16 and 35 regularly visited the ED in both CTAS levels [14]. Additionally, 34.4% of the cases in this study were older than 65. Older patients were reported to be more likely to attend the ED because they believed it provided better care and investigation access. Furthermore, it may reflect the burden of chronic disease in this age group and the necessity for expert treatments [15].

Our data show that most ED visitors were women (54.1%). A systematic evaluation of improper use of emergency services indicated that women have greater odds of incorrectly visiting the ED [23]. A similar result was found in a study by Alnasser et al. in which 64.5% of cases were female [14]. Women were more likely to visit the ED due to ease and accessibility. In Saudi Arabia, women usually cannot travel alone without a male "mahram" family member, and they frequently have to wait for a male family member to transport them to the ED after work [15].

This study's leading ED delay causes were multiple consultations with further investigations (37.7%) and conflict between the teams (36.1%). Similarly, a Dutch study indicated that organizational characteristics such as more consultations, testing in the ED, and lower physician seniority were associated with prolonged lengths of stay in the ED [8]. ED physicians, like other specialists, may request consults for non-mandatory reasons. However, they require further consultations for operations needing specialized knowledge or for in-hospital admission. Furthermore, patients referred to emergency departments usually had non-specific symptoms and were more likely to come with undifferentiated concerns [24,25]. Based on univariate analysis, we found that multiple consultations with further investigation requests are predictors of delays in ED. Similarly, Busti et al. and Lee et al. highlight the impact of a high number of consultations on ED length of stay and recommend strategies to reduce this impact [4,26].

Furthermore, we found that multiple assessments in different ED areas with delayed arrival of a specialist were predictors of ED delays. Workload balancing is an essential concern in emergency department physician rostering. Efficient scheduling and workload distribution among physicians can help maximize performance and resource use [27]. A study by van der Veen et al. found that facilitating timely consultation, i.e., shortly after triage, might be another strategy to lessen the influence of consultation on ED length of stay [24]. In a study from Canada, ED patients referred to another specialty were older, had greater acuity presentations, and arrived by ambulance more frequently than patients without consultation [28]. Similar patient characteristics were independent predictors of consultations in the current study, indicating that they are universal across healthcare systems [24].

In this study, the team conflict was presented in 36.1% and was a predictor for ED delay in univariate analysis (OR: 2.50, p= 0.031). Incompatible personal motives, a heavy workload, stress, role ambiguity, and poor leadership all contribute to conflict in the healthcare field [26,29]. According to narrative analysis, conflict was frequently observed during referrals or admissions from ED to inpatient or admitting units. Individual-level causes of conflict include a lack of faith in the ED workup and professional inexperience [30]. Perceptions of prejudice between groups, patient complexity, communication failures, and differences in practice are all factors that contribute to team performance [30]. Van der Veen et al. found that 24% of 1434 ED visits received an additional specialist consultation, resulting in a 55% increase in ED duration of stay. However, the majority of this session was required to determine the patient's treatment plan, which included admission or discharge with outpatient follow-up [24]. A recent study found that conflicts in medical teams are caused by a high workload, resource usage, inaccurate evaluations, and an inadequate supply of resources [31]. Evidence-based frameworks such as the 5Cs of Consultation, PIQUED, and SBAR seek to improve communication between physicians during consultations. However, these models frequently ignore consultant physicians' activities, instead focusing on consultation request structure and referring physician behavior [32]. Consultants' commandments emphasize determining consultation issues, urgency, acquiring primary data, offering clear communication, making explicit suggestions, clarifying roles, training referring physicians, and communicating directly. These proposals were influential; however, they were based on opinion and were intended for asynchronous consultations. Technological advancements have resulted in greater real-time, simultaneous communication and increased consultant skills, hence boosting consult volumes and needs [33]. Better communication training, standardizing admission rules, and enhancing interdepartmental connections have all been mentioned as potential dispute resolution strategies [30].

In this study, in the ER setting, 37.7% of cases received appropriate and important further investigations, while 9.83% were deemed inappropriate and unimportant. Balance measures to consider when ordering tests at triage based on minimal history and exam, whether through triage nursing orders or providers in triage, include the percentage of tests ordered at triage that prove to be clinically irrelevant, along with the percentage of patients who have a separate diagnostic work‐up initiated by the treating clinician because the initial work‐up was insufficient or inappropriate (thus paradoxically negating the benefit of earlier resulting of those tests, and ultimately leading to over‐testing) [34]. An alternative hypothesis is that providers in triage could reduce unnecessary testing compared to triage nursing orders to the extent that triage providers place more targeted orders [35]. In another report, Kaushik et al. found a positive correlation between laboratory turnaround time and ED length of stay, with a one-minute decrease in turnaround time resulting in a 0.50-minute decrease in length of stay. Turnaround time reductions of 5-, 10-, and 15 minutes could potentially admit 127, 256, and 386 additional patients annually, respectively [36].

In this study, we found 63.9.1% agreement between emergency physicians and consultants on admission needs, with 63.3% of patients receiving one consultation and 37.7% receiving multiple consultations, with 7.37% deemed difficult by emergency physicians. In a previous report, Woods et al. showed that consultation is a crucial aspect of ED care, with 38% of patients requesting a consultation. Over half of these patients (54.3%) were admitted to the hospital. Consultation proportions were similar between males and females, but more frequent for older patients, higher acuity presentations, daytime hours, or ambulance arrivals. The proportion of agreement between emergency physicians and consultant opinions on admission needs was 89%. Around 92% of patients received one consultation, with 6% perceived as difficult by emergency physicians [28]. In another report, 27 clinicians and managers supported a two-hour Board Round in ED injuries departments. A multi-disciplinary meeting led by the lead nurse with support from the emergency physician in charge was preferred. The Path-Goal Leadership survey instrument revealed a highly directive leadership style [37].

Study limitations

This study has been limited by its retrospective methodology and small sample size, which assessed the electronic medical records of all patients who attended the King Khalid Hospital in Najran, Saudi Arabia. The utilization of a retrospective document review for patients' ultimate diagnosis may be unrelated to their principal complaint or original triage level. This study was also done during the COVID-19 pandemic; therefore, it may not accurately reflect the trend of frequent ED visits. This research's generalizability may be restricted because it is a single-center study. In addition, further comparisons and statistical testing should be undertaken in subsequent investigations. CTAS was used to triage ED visits subjectively based on the triage nurse's assessment. However, the findings of this study might be utilized to build rules governing the proper usage of primary healthcare institutions.

## Conclusions

In this study, multiple consultations with further investigations, various assessments in different ED areas with the late arrival of the specialist, and conflict between the teams were the main factors for delays in ED. Understanding teamwork collaboration and implementing a timeframe monitoring system for consultations while emphasizing accelerated decision-making and disposition status of patients can reduce the ED length of stay in ED. Implementing these strategies and evaluating their impact on the length of stay in the ED requires further investigation.
